# Challenges of pain management in neurologically injured patients: systematic review protocol of analgesia and sedation strategies for early recovery from neurointensive care

**DOI:** 10.1186/s13643-018-0756-z

**Published:** 2018-07-24

**Authors:** David Wyler, Michael Esterlis, Brittany Burns Dennis, Andrew Ng, Abhijit Lele

**Affiliations:** 1 Department of Anesthesiology and Pain Medicine, Thomas Jefferson University, 111 South 11th Street Suite 8490 Gibbon, Philadelphia, PA 19107 USA; 2 Department of Critical Care Medicine and Neurological Surgery, Thomas Jefferson University, 111 South 11th Street Suite 8490 Gibbon, Philadelphia, PA 19107 USA; 3 Department of Anesthesia, University of Toronto, Toronto, Canada; 4 St George’s University of London Medical School, London, UK; 5 Department of Anesthesiology and Pain Medicine, University of Washington, Harborview Medical Center, Seattle, USA

**Keywords:** NeuroICU, Neurointensive care, ICU, Intensive care unit ABCDEF, Pain assessment, Analgesia, Sedation, Pain management protocol, Ventilation weaning, Spontaneous awakening trials, Spontaneous breathing trials, Early mobility, Liberation, Animation, Family engagement and empowerment

## Abstract

**Background:**

A recent paradigm shift within the intensive care discipline has led to implementation of protocols to drive early recovery from the intensive care unit (ICU). These protocols belong to a large knowledge, translation and quality improvement initiative lead by the Society of Critical Care Medicine, aiming to “liberate” patients from the ICU. They “bundle” evidence-based elements shown to lower ICU stay and mortality and optimize pain management. The bundled elements focus on Assessing, preventing and managing pain; Both spontaneous awakening trials and spontaneous breathing trials; Choice of analgesia and sedation; assessment, prevention, and management of Delirium; Early mobility and exercise; and Family engagement and empowerment (ABCDEF). It is evident that analgesia and sedation protocols either directly relate to or influence most of the bundle elements. A paucity of literature exists for neurologically injured patients, who create unique challenges to bundle implementation and often have limited external validity in existent studies. We will systematically search the literature, present the unique challenges of neurointensive care patients, conduct a stratified analysis of subgroups of interest, and disseminate the evidence of analgesia and sedation protocols in the neurointensive care unit (NICU). We hope the relevant stakeholders can adapt this information through knowledge translation—to make formal recommendations in clinical practice guidelines or a position statement.

**Methods/design:**

The authors will search MEDLINE (PubMed), EMBASE, Cochrane Library, Cochrane Clinical Trials Registry, World Health Organization International Clinical Trials Registry Platform Search Portal, and the National Institutes for Health Clinical Trials Registry. The title, abstract, and full-text screening will be completed in duplicate, and a Cohen’s Kappa coefficient of agreement will be reported. Provided the data retrieved from studies is suitable, results will be combined statistically using meta-analysis. We aim to evaluate the impact of ABCDEF bundle components on multiple endpoints of NICU recovery. Our primary outcomes will be time to successful discontinuation of mechanical ventilation and time to early mobility. The authors will guide the methodological design of the study using the PRISMA-statement and the checklist compliance will be available.

**Discussion:**

Using the evidence from this systematic review, we anticipate disseminating knowledge of analgesia and sedation protocols in the NICU. The results of this systematic review are imperative to close the knowledge gap in a patient population that is often excluded from studies, and to add to the body of literature aiming to enhance early recovery from the NICU and mitigate iatrogenic harm.

**Systematic review registration:**

PROSPERO CRD42017078909

## Background

There is a growing body of knowledge that contributes to our understanding of the devastating outcomes of critical illness [1]. It is through such rigorous investigations that we learned our iatrogenic contribution to patient morbidity and mortality. A prominent example is unoptimized mechanical ventilation up until the 1990s and the development of the ARDSnet protocol [2, 3] which has since saved thousands of lives of those mechanically ventilated in the intensive care unit (ICU). A clinical area left largely unexplored is analgesia and sedation practices in the ICU. Although the practice has evolved in the last decade, many of our current clinical decisions are not strongly supported by high quality (level 1) evidence [4]. The paucity in evidence could be explained by difficulty in trial design, simultaneous use of several pharmacologic agents leading to a confounding effect, and heterogeneity in ICU patient populations leading to limited external validity [4]. Sedation practices have been shown to influence extubation and mortality [5]. In light of our growing understanding of analgesia and sedation practices contributing to iatrogenic harm, current practices and their outcomes have been the subject of a large body of research. One complication that has been shown to increase morbidity is ICU-related cognitive impairment [6, 7]. Certain risk factors for this ICU-acquired phenomenon that has been termed “post-intensive care syndrome” (PICS) include delirium [8], pain, and agitation (PAD), which are commonly experienced [9–11]. Furthermore, pain has been shown to be the largest concern of patients, and its recall has been associated with post-traumatic stress disorder, chronic pain, and reduced quality of life after discharge. With more than 4 million ICU admissions per year [8], the overall impact on those patients who survive ICU stay can be significant. ICU stay has been associated with long-term cognitive, psychological, neuromuscular, and functional deficits all of which contribute to PICS, which can leave patients with a significantly impaired quality of life post-discharge [12–16].

Pain was once considered the “fifth vital sign,” and this approach has been recently scrutinized in light of the growing concern of the modern opioid epidemic and fatal respiratory depression events attributed to liberal sedation practices [17]. The Joint Commission on Accreditation of Healthcare Organizations (JCAHO) recommendations for pain assessment and treatment in hospitalized patients [17, 18] were revised since their inception in 2000, with the latest iteration in 2017. The current standards advocate for multi-modal analgesic regimens. These regimens include setting realistic pain expectations, identifying psychosocial factors that may affect self-report of pain, rigid prescription accountability, vigilant monitoring of high-risk patients, and implementation of prescription drug monitoring programs [19]. In 2013, the American College of Critical Care Medicine revised its 2002 clinical practice guidelines of pain, agitation, and delirium (PAD) of adults in the ICU to reflect ‘analgosedation’ sparing practices [20]. There is new evidence to support the paradigm shift of minimizing analgosedation in the ICU. One parallel-design RCT [21] demonstrated that immediate sedation interruption in the ICU resulted in statistically and clinically significant expedited extubation, reduced time on mechanical ventilation, delirium, and coma.

Efforts by the Society of Critical Care Medicine aim to optimize pain management while reducing delirium and long-term adverse consequences of ICU admission. This quality improvement and knowledge translation initiative known as “ICU liberation” sets its foundation on “bundling” several elements shown to expedite mechanical ventilation weaning and encourage early mobility [13]. These bundled elements focus on Assessing, preventing, and managing pain; Both spontaneous awakening trials and spontaneous breathing trials; Choice of analgesia and sedation; assessment, prevention and management of Delirium; Early mobility and exercise; Family engagement and empowerment (ABCDEF). There is an abundance of evidence to support spontaneous awakening and breathing trials [22–25]; provide light sedation to maintain patients at an awake and alert level and avoidance of benzodiazepines [26–31]; assess, prevent, and manage delirium [32–36]; encourage early mobility and exercise [37–41] (a dogma that was once implemented, abandoned, and now rediscovered [13]); and engage and empower families [42–44].

A cross-sectional study [45] with 143 mechanically ventilated patients in a single-center ICU compared participants with bundle implementation (A through E) [*n* = 73] to those without [*n* = 70]. The authors found a statistically significant [*p* < 0.05] improvement in hemodynamics at 3, 5, and 7 days after bundle implementation (mean arterial pressure, central venous pressure, heart rate), oxygenation index (PaO2/FiO2), reduced requirement of Sufentanil and Midazolam, reduced delirium, improved 28-day survival, and reduced mechanical ventilation duration and total ICU length of stay. Despite the strong evidence advocating for ABCDEF bundle implementation, there is still low compliance amongst international ICUs. A large international survey [46], with 1521 ICUs respondents across 47 countries, showed that only 57% reported to implement the ABCDEF bundle. A recent large cohort study that surveyed six ICUs and 6064 patients (of which 1438 received mechanical ventilation and thus the ‘full ABCDEF bundle implementation’) [1] demonstrated a statistically significant reduction of delirium and mortality with bundle implementation (a 15% higher hospital survival for every 10% increase in partial bundle compliance). Average ICU mortality in adults, estimated at 10–29%, varies according to population traits such as age, comorbidities, and illness severity [47]. There seems to be a dose-response relationship between bundle compliance and favorable patient outcomes such as reduced ICU mortality and increased hospital survival. The aforementioned study [1] is the largest we are aware of that directly measures cohort outcomes of ‘pre- and post-ABCDEF bundle implementation’ and thus offers quantitative data for its benefits.

It is clear that the choice of analgesic modality influences most bundle elements. For example, several pharmacological elements indicated for analgesia may also confound respiratory drive, mechanical ventilation weaning, development of delirium, and ability to mobilize early. A group that is often excluded from studies is neurologically injured adults [48], thus limiting the generalization of the results and threatening the external validity in the NICU. Guidelines exclude neurologically injured patients for safety reasons [20]. The most obvious safety concern arises in the setting of high intracranial pressure (ICP). Moreover, in the NICU, sedatives and analgesics serve as therapy to lower ICP, prevent brain compression and subsequent herniation. Additionally, patients with raised ICP are not candidates for a spontaneous awakening and breathing trials, as these maneuvers can further exacerbate raised ICP. Analgesia and sedation practices indeed create a unique challenge in the NICU setting as it may influence the neurological exam, and arousal in patients whose central nervous system has already been injured. An important component of neuromonitoring in the NICU is the gold-standard serial neurological wake-up tests [49]. Other challenges include balancing a neurological exam versus optimizing neurologic parameters such as cerebral blood volume, ICP, cerebral metabolic rate for oxygen and seizure control both in terms of prophylaxis or treatment [18, 48, 50, 51]. Additionally, an underlying neurological condition may affect patients’ ability to communicate, mobilize, breathe, and impact their pain threshold, all of which are directly related to and influence liberation from the NICU.

We identified a paucity of literature with respect to neurologically injured adults receiving intensive care, which has resonated in earlier reports [9]. Additionally, no other study to our knowledge explored challenges to ABCDEF bundle implementation in the NICU. Furthermore, a recently published international survey and practice audit of six NICUs showed discordance in physician self-reporting, and thus analgesia regimens require further work for optimization [52]. This systematic review will investigate the components of the ABCDEF bundle as they are applied to neurologically injured adults and explore evidence of challenges experienced in analgosedation practices in the NICU.

### Objectives

This systematic review aims to identify the challenges of analgesia and sedation in the context of the ABCDEF bundle implementation in the NICU. The only systematic review we found that addressed barriers to ABCDEF implementation [53] was not specific to the unique considerations of neurologically injured adults. Provided that most studies exclude this patient population, a study that elucidates the external validity of bundle implementation to the NICU is warranted. Furthermore, our goal is to “unbundle” the liberation components that older studies might have addressed individually and pool their results.

Specifically, the objectives of this investigation include:

1. Assessing the transferability of ABCDEF bundle components to patients admitted to the NICU
2. Compare the efficacy of different analgesic and sedation modalities across ventilation weaning, early mobilization, and post-discharge outcomes amongst patients admitted to NICU
3. Determine whether particular analgesic and sedation agents or regimens optimize outcomes in patients admitted to the NICU when stratified for various neurological disorders
4. Critically evaluate the current literature and identify important knowledge gaps that future research should address
5. Offer evidence to be used for knowledge translation by relevant stakeholders
6. Publish the final report in an open access journal to eliminate restrictions to knowledge dissemination

We are interested in the analgesia and sedation regimens in the NICU, and hope the preliminary findings of this systematic review will set the groundwork to further clinical questions and research addressing each component of the ABCDEF bundle.

### Research question

In the neurologically injured adult receiving neurointensive care, does implementation of the ABCDEF bundle or its individual parts, improve time to wean off mechanical ventilation, time to mobilization, and reduce long-term complications associated with intensive care therapy?

Please refer to Table 1 for the PICO question used to derive our research question.

**Table 1:** PICO question used to derive the research question

Population: neurologically injured adults (> 18 years-old), admitted to the NICU secondary to traumatic brain injury, stroke (ischemic or hemorrhagic), or postoperative cranial or spinal surgery with or without coexisting neurodegenerative disease.	
Intervention: implementation of parts or entirety of the ABCDEF bundle or *other pain management strategies*	
Comparison: no implementation of any ABCDEF bundle parts	
Outcomes: primary outcomes: (1) time to wean off mechanical ventilation, (2) time to early mobilization	
Secondary outcomes: incidence of cognitive and psychological long-term outcomes (post-intensive care syndrome), time to discharge, frequency of dosing of opioids (patient-controlled analgesia, as needed dosing, or scheduled dosing), frequency of any medication administration with the primary indication of sedation, incidence of agitation and delirium, and incidence of opioid addiction after discharge.	

## Methods/design

We will conduct a comprehensive search of the available literature. In order to broaden our capture strategy, we will not include outcomes (e.g., mortality, post-intensive care syndrome) in our search strategy and rather screen for these outcomes during the study extraction process with an identical checklist that will be developed by consensus of the two screeners (ME and BD) and approved by all the remaining of the authors. We will search the following online databases: MEDLINE (Ovid)/PubMed, EMBASE (Ovid), Cochrane Library, Cochrane Clinical Trials Registry, World Health Organization (WHO) International Clinical Trials Registry Platform Search Portal, and the National Institutes for Health (NIH) Clinical Trials Registry. We will use a university library subscription accessed through (library.sgul.ac.uk/). The search terms were agreed upon by consensus, and MeSH and Emtree terms were included for completeness and to broaden the search in case articles were incompletely indexed by the databases. The team agreed it was preferable to have redundant search terms to broaden the search and narrow the study selection during the screening phase. Searches will be performed independently by two authors (ME and BD). We will not be searching the gray literature with the exception of official material published on the Society of Critical Care Medicine ICU Liberation website (http://www.iculiberation.org). We will also contact each primary investigator listed on the NIH Clinical Trial Registry from studies deemed eligible during the title screening, where we will inform the investigators of our systematic review and ask for information regarding any publications resulting from their trial. We consulted a librarian from the Scott Memorial Library of Thomas Jefferson University with expertise in systematic reviews to assist with the process of devising the search strategy and conduct the literature search. The two authors (ME and BD) will then independently manually scan the bibliography of all studies that met the inclusion criteria to ensure no relevant titles were missed. Only studies published in the English language will be extracted. We will constrain the search for studies published after 1992. Only human studies will be included. We will also eliminate incomplete studies, as they would not provide sufficient data for extraction. We will inform the authors of the eligible articles about the review during the data extraction process to consult them for clarification of their data when needed. Please refer to Table 2 for the full search strategy, which may be subject to minor revisions for the final systematic review.

**Table 2:** Defined search strategy for the extraction of pertinent studies from multiple databases

EMBASE search strategy search = ____	1. (Neuro$ ICU or Neuro$ Intensive Care or Neurocritical care or intensive care or critical care or ICU).mp. or Intensive care units/ [mp = title, abstract, original title, name of substance word, subject heading word, keyword heading word, protocol supplementary concept word, rare disease supplementary concept word, unique identifier, synonyms] 2. (Brain Injury or TBI or neuro$ trauma or neuro$ injury or cerebral injury or spin$ injur$).mp. or Spinal Cord Injuries/ or Brain Injuries/[mp = title, abstract, original title, name of substance word, subject heading word, keyword heading word, protocol supplementary concept word, rare disease supplementary concept word, unique identifier, synonyms] 3. (Stroke or Intracranial hemorrhage or intracranial hemorrhage or Subarachnoid hemorrhage or Subarachnoid hemorrhage or CVA or ischemic stroke or ischaemic stroke or Cerebral clot or Cerebral thrombosis).mp. or Nervous system diseases/ or Brain neoplasms.mp. or Cerebrovascular Disorders/ or Cerebral Hemorrhage/ or Cerebral Infarction/ or spinal diseases/ [mp = title, abstract, original title, name of substance word, subject heading word, keyword heading word, protocol supplementary concept word, rare disease supplementary concept word, unique identifier, synonyms] 4. ((((Cranial and surg$) or Cranial) and opera$) or Craniotomy).mp. or Neurosurgical procedures/ [mp = title, abstract, original title, name of substance word, subject heading word, keyword heading word, protocol supplementary concept word, rare disease supplementary concept word, unique identifier, synonyms] 5. (((Spin$ and surgery) or Spin$) and opera$).mp. [mp = title, abstract, original title, name of substance word, subject heading word, keyword heading word, protocol supplementary concept word, rare disease supplementary concept word, unique identifier, synonyms] 6. (((liberation or ABCDE$ or Awakening) and breathing coordination) or Delirium monitor$).mp. or Delirium/ or exercise.mp. or mobility.mp. or animation.mp. or analgesia.mp. or Analgesia/ or pain management.mp. or Pain Management/ or sedation.mp. or assessment.mp. or neuro$ exam.mp. or challenge$.mp. or family.mp. or Family/ [mp = title, abstract, original title, name of substance word, subject heading word, keyword heading word, protocol supplementary concept word, rare disease supplementary concept word, unique identifier, synonyms] 7. 2 or 3 or 4 or 5 8. 1 and 6 and 7 9. limit 8 to (human and english language and yr. = “1992 -Current”)
Medline (PubMed) search strategy search = ____	1. (Neuro* ICU or Neuro* Intensive Care or Neurocritical care or intensive care or critical care or ICU).mp. or Intensive care units/[mp = title, abstract, original title, name of substance word, subject heading word, keyword heading word, protocol supplementary concept word, rare disease supplementary concept word, unique identifier, synonyms] 2. (Brain Injury or neuro* trauma or neuro* injury or cerebral injury or spin* injur*).mp. or Spinal Cord Injuries/ or Brain Injuries/ [mp = title, abstract, original title, name of substance word, subject heading word, keyword heading word, protocol supplementary concept word, rare disease supplementary concept word, unique identifier, synonyms] 3. (Stroke or Intracranial hemorrhage or intracranial hemorrhage or Subarachnoid hemorrhage or Subarachnoid hemorrhage or CVA or ischemic stroke or ischaemic stroke or Cerebral clot or Cerebral thrombosis).mp. or Nervous system diseases/ or Brain neoplasms.mp. or Cerebrovascular Disorders/ or Cerebral Hemorrhage/ or Cerebral Infarction/ or spinal diseases/ [mp = title, abstract, original title, name of substance word, subject heading word, keyword heading word, protocol supplementary concept word, rare disease supplementary concept word, unique identifier, synonyms] 4. ((((Cranial and surg*) or Cranial) and opera*) or Craniotomy).mp. or Neurosurgical procedures/ [mp = title, abstract, original title, name of substance word, subject heading word, keyword heading word, protocol supplementary concept word, rare disease supplementary concept word, unique identifier, synonyms] 5. (((Spin* and surgery) or Spin*) and opera*).mp. [mp = title, abstract, original title, name of substance word, subject heading word, keyword heading word, protocol supplementary concept word, rare disease supplementary concept word, unique identifier, synonyms] 6. (((liberation or ABCDE* or Awakening) and breathing coordination) or Delirium monitor*).mp. or Delirium/ or exercise.mp. or mobility.mp. or animation.mp. or analgesia.mp. or Analgesia/ or pain management.mp. or Pain Management/ or sedation.mp. or assessment.mp. or neuro* exam.mp. or challenge*.mp. or family.mp. or Family/ [mp = title, abstract, original title, name of substance word, subject heading word, keyword heading word, protocol supplementary concept word, rare disease supplementary concept word, unique identifier, synonyms]
“term”/=Emtree or MeSH term	7. 2 or 3 or 4 or 5 8. 1 and 6 and 7 9. limit 8 to (human and english language and yr. = “1992 -Current”)
Cochrane Library search strategy search = ____	1. Search title, abstract, keywords: ABCDEF 2. Search title, abstract, keywords: ABCDE 3. Search title, abstract, keywords: Neurointensive 4. Search title, abstract, keywords: ICU 5. Search title, abstract, keywords: Intensive Care
World Health Organization (WHO) International Clinical Trials Registry Platform Search Strategy search = ____	Advanced Search: [‘In the title’] ABCDE OR ABCDEF OR intensive* OR ICU OR critical care OR Neurocritical care OR intensive care AND [‘In the Intervention’] analgesia OR pain OR opioid OR sedation OR mobility OR weaning OR animation OR bundle OR family
Clinical Trials Registry (through National Institute for Health) search strategy search = ____	Advanced Search: ‘other terms’: (ABCDE OR ABCDEF OR intensive* OR ICU OR critical care OR Neurocritical care OR intensive care) AND (analgesia OR pain OR opioid OR sedation OR mobility OR weaning OR animation OR bundle OR family)

### Selection of studies

**Fig. 1:**
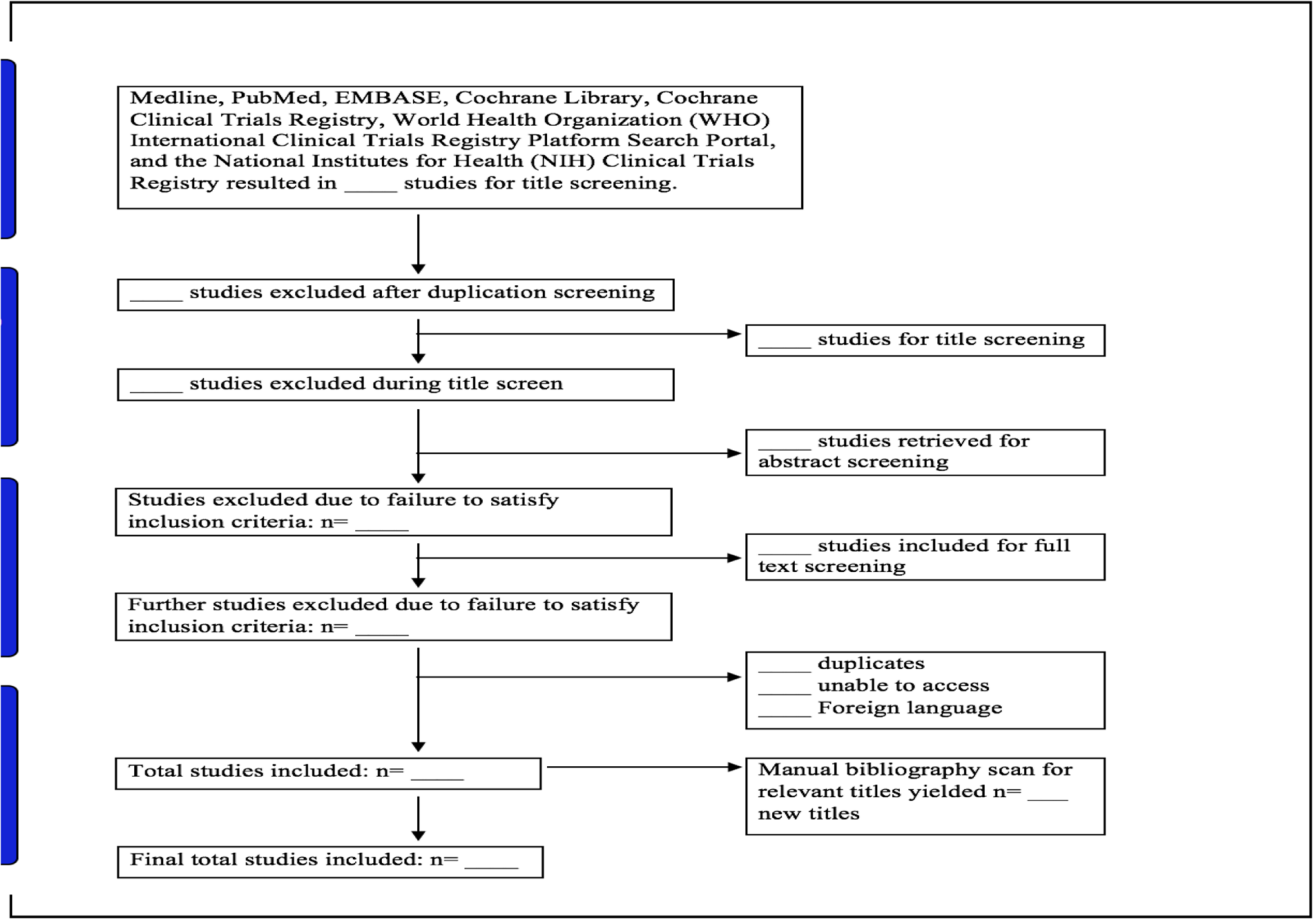
Flow diagram of screening process

The authors (ME and BD) will independently conduct a primary title search, title screening, abstract screening, and full-text extraction. We will refer to the inclusion and exclusion criteria throughout the screening process and reject articles that are not relevant. We will utilize the DistillerSR (https://www.evidencepartners.com/products/distillersr-systematic-review-software/) to screen titles and abstracts extracted across all the database searches. In the case of a disagreement during the search and selection process, we will engage in discussion to reach a consensus. Should a conflict persist, a third author (DW) will facilitate the resolution. Agreement level between reviewers will be assessed using the Cohen’s Kappa coefficient. As per guidelines set by the Preferred Reporting Items for Systematic Reviews and Meta-analyses (PRISMA), a flow diagram will be included to display the screening process (Fig. 1) and a detailed table of the studies selected in the systematic review [54].

### Inclusion and exclusion criteria

The authors will limit the studies to be included in this review to randomized controlled trials (RCTs) and high-quality observational (prospective or retrospective-cohort) studies evaluating the implementation of any of the ABCDEF bundle elements (either individually or together) in the NICU. Specifically, the study would have had to examine *any* of the following in adults (≥ 18 years old) in the context of a neurological injury. (1) *assessment* of pain management, (2) spontaneous awakening trials and/or spontaneous breathing trials, (3) analgesia and sedation (4) delirium, (5) early mobility and/or exercise, (6) family engagement and/or empowerment. The study participants may be spontaneously breathing, assisted, or mechanically ventilated, receiving any form of analgesia with or without concomitant use of agents primarily indicated for sedation, and with or without use of alternative non-pharmacological therapies. The PRIMARY outcomes are measured in a unit of time. We will define wean as a successful cessation of mechanical ventilation for more than 24 h. While mobility or animation has been defined as “getting patients out of bed” and walking (even if intubated and mechanically ventilated) [13], this approach of *active patient engagement* does not necessarily capture the challenges of patients with neurological injury, whose paresis, paralysis, spasticity, and neuropathic pain may impede on this goal. We will define mobilization as any attempt to engage in assisted walking or physical exercise or physical therapy such as passive range of motion exercises. Patients in the NICU may indeed benefit from passive range of motion as therapy for contractures, pain, and enhance their probability to eventual ‘active mobility.’

We will set exclusion criteria to control for bias and confounding variables such as a severe neurological injury that prevents outcome measures (e.g., irreversible neurological injury or persistent vegetative state), expected mortality < 7 days or palliative care, a severe baseline (pre-NICU admission) neurocognitive deficit (such as dementia or a severe intellectual disability). All studies must be primary investigations with comparison groups. We will not include pilot studies or RCTs at phases 0, 1, and 2. Case reports and case series will be excluded from our review due to their low external validity. All studies selected for inclusion into our manuscript will be required to demonstrate an ethics review board approval in accordance with the Helsinki Declaration.

### Statistical analysis plan and quality assessment of individual studies

Results from this review will be summarized both narratively and statistically using meta-analysis where possible. When statistical pooling of results from included studies is not feasible, we will summarize our findings narratively by reporting the individual descriptive statistics from included studies. Summary estimates will be calculated using the pooled standardized mean differences for continuous outcomes and pooled odds ratios for dichotomous outcomes. All summary estimates will be presented in forest plots. Due to the anticipated heterogeneity in the study populations, a random-effect meta-analysis with a DerSimonian Laird estimator will be used. All studies will be weighted according to the inverse of the variance. As it is highly cautioned against to pool experimental (randomized) and non-randomized studies, we will require studies to share the same research methodology. In efforts to reduce the impact of selection bias on the pooled estimates, we will require studies to share the same research design (e.g., RCT) when included in the meta-analysis [55, 56].

Provided an appropriate number of studies are eligible for inclusion, we will assess for publication bias using an Egger’s plot.

We will rely on the *I*^2^ statistic to interpret the level of heterogeneity affecting the pooled meta-analysis findings. To interpret the *I*^2^, we will use thresholds set forth by the Cochrane Collaboration, these include *I*^2^ of 0–40% (might not be important), 30–60% (moderate heterogeneity), 50–90% (substantial heterogeneity), and 75–100% (considerable heterogeneity) [55].

The differences in the patient population as well as methodological quality are anticipated to be important factors for explaining heterogeneity between studies. Provided we have a suitable number of studies, we will conduct subgroup analyses (whenever data is available) to assess the robustness of our meta-analysis findings when stratified by (1) methodology quality based on the risk bias assessment scores, (2) category of neurological disorder, injury, or admission diagnosis to the NICU, (3) neurological injury assessment tools such as the Glasgow Coma Scale, (4) whether the patients received mechanical ventilation, (5) BMI, (6) age, (7) baseline opioid usage and indication. The differences in methodological quality will be captured using the scores obtained from the risk of bias assessment, using the modified Newcastle-Ottawa scale for observational studies and the Cochrane Risk of Bias tool for RCTs. Utilizing the scores attained during the risk of bias assessment, studies will be categorized into high or low methodological quality using the standard methodological scoring cut-offs used in previous reviews [57]. We anticipate that the implementation of optimal analgesia and sedation regimens within different subgroups in the NICU to differ for a multitude of reasons, and therefore, it is important to compare the statistical outcomes of subgroups of patients receiving neurointensive care. First, patients receiving mechanical ventilation are by definition of those who we are seeking to ‘liberate’ from the NICU. Comparing them to those who are not mechanically ventilated can shed light into optimizing analgesia and sedation regimens specific to this subgroup. Patient characteristics such as age, BMI, and baseline opioid usage may affect opioid sensitivity and may warrant their stratified analysis for the primary and secondary outcomes. Furthermore, we will critically examine whether a malignancy diagnosis poses challenges to analgesia and ICU liberation as there are unique consideration when treating “cancer pain.” This may provide new insight into challenges of particular patient populations and pave the path to new research to assist in their recovery from the NICU.

All analyses will be performed using STATA Version 13.

### GRADE framework

We will assess the quality of evidence using the Grades of Recommendation, Assessment, Development, and Evaluation (GRADE) framework [58]. Most importantly, we will scrutinize the quality of evidence as has been described in the original publications from the GRADE Working Group [59]. We will include a reference table in our proposed review for readers to easily visualize what the different quality measurements that we refer to are defined by.

## Discussion

There are some concerns with respect to the choice of analgesic modalities in the NICU. Opioid addiction has been shown to be as high as 44% in those receiving long term infusions [60]. Strikingly, opioids have been found to be prescribed in 97% of NICU patients [52]. Although these results stem from a small study sample of 173 patients [52], and the methodological limitations of an observational study, they call for research that can provide a high-quality evidence to guide future analgosedation practices in the NICU. We aim to fill the current paucity in the literature as it pertains to a unique patient population. We believe our outcomes will be finally generalizable to a patient population that has been traditionally excluded from studies and guidelines [20, 48]. The dissemination of our objective review will be imperative to enhance clinical practice and evaluate the different components of the ABCDEF bundle, and hopefully to quantify the impact of bundle implementation to the various outcomes we have described. Some bundle components have been shown to have an economic benefit [61], and as researchers contribute to the collective growing body of knowledge, we anticipate that changes in practice will soon become the standard of care.

## Abbreviations

ABCDEF: Assessing, preventing, and managing pain; Both spontaneous awakening trials and spontaneous breathing trials; Choice of analgesia and sedation; assessment, prevention, and management of Delirium; Early mobility and exercise; Family engagement and empowerment
ICP: IntraCranial pressure
ICU: Intensive care unit
JCAHO: Joint Commission on Accreditation of Healthcare Organizations
NICU: Neurointensive care unit
PRISMA: Preferred Reporting Items for Systematic Reviews and Meta-analyses
RCT: Randomized control trial
WHO: World Health Organization
